# Subjective and objective responses to caloric stimulation help separate vestibular migraine from other vestibular disorders

**DOI:** 10.1007/s00415-023-12027-z

**Published:** 2023-10-17

**Authors:** I. P. Hannigan, S. M. Rosengren, G. K. Bharathy, M. Prasad, M. S. Welgampola, S. R. D. Watson

**Affiliations:** 1 Central Clinical School, Faculty of Medicine and Health, University of Sydney, Sydney, 2006 Australia; 2 Institute of Clinical Neurosciences, Royal Prince Alfred Hospital, Camperdown, 2006 Australia; 3 School of Computer Science, Faculty of Engineering and Information Technology, University of Technology Sydney, Sydney, 2007 Australia; 4 Prince of Wales Clinical School, University of New South Wales, Randwick, 2013 Australia; 5 Blacktown Neurology Clinic, Blacktown, NSW 2148 Australia

**Keywords:** Vestibular migraine, Caloric test, Subjective vertigo

## Abstract

**Background:**

Nystagmus generated during bithermal caloric test assesses the horizontal vestibulo-ocular-reflex. Any induced symptoms are considered unwanted side effects rather than diagnostic information.

**Aim:**

We hypothesized that nystagmus slow-phase-velocity (SPV) and subjective symptoms during caloric testing would be higher in vestibular migraine (VM) patients compared with peripheral disorders such as Meniere’s disease (MD) and non-vestibular dizziness (NVD).

**Methods:**

Consecutive patients (*n* = 1373, 60% female) referred for caloric testing were recruited. During caloric irrigations, patients scored their subjective sensations. We assessed objective-measures, subjective vertigo (SVS), subjective nausea (SNS), and test completion status.

**Results:**

Nystagmus SPV for VM, MD (unaffected side), and NVD were 29 ± 12.8, 30 ± 15.4, and 28 ± 14.2 for warm irrigation and 24 ± 8.9, 22 ± 10.0, and 25 ± 12.8 for cold-irrigation. The mean SVS were 2.5 ± 1.1, 1.5 ± 1.33, and 1.5 ± 1.42 for warm irrigation and 2.2 ± 1.1, 1.1 ± 1.19, and 1.1 ± 1.16 for cold-irrigation. Age was significantly correlated with SVS and SNS, (*p* < 0.001) for both. The SVS and SNS were significantly higher in VM compared with non-VM groups (*p* < 0.001), and there was no difference in nystagmus SPV. VM patients SVS was significantly different to the SVS of migraineurs in the other diagnostic groups (*p* < 0.001). Testing was incomplete for 34.4% of VM and 3.2% of MD patients. To separate VM from MD, we computed a composite value representing the caloric data, with 83% sensitivity and 71% specificity. Application of machine learning to these metrics plus patient demographics yielded better separation (96% sensitivity and 85% specificity).

**Conclusion:**

Perceptual differences between VM and non-VM patients during caloric stimulation indicate that subjective ratings during caloric testing are meaningful measures. Combining objective and subjective measures could provide optimal separation of VM from MD.

**Supplementary Information:**

The online version contains supplementary material available at 10.1007/s00415-023-12027-z.

## Background

Robert Barany was awarded the Nobel Prize in physiology or medicine in 1914, in large part for his characterization of caloric nystagmus and head position on the caloric response [1]. Then, Fitzgerald and Hallpike introduced the popular clinical bithermal caloric test [2]. Until recent years, this test has been the only clinical means for testing the integrity of the vestibular labyrinth of one ear, in isolation from the contralateral labyrinth. Today, in the era of head impulse and vestibular evoked myogenic potentials, the caloric-evoked vestibulo-ocular reflex (VOR) test continues to have an important clinical role in most vestibular clinics.

In addition to the quantitative VOR-mediated eye movement, the common measure of interest with the caloric test, there is often an induced vertigo, nausea, and unpleasant sensations. Although at times limiting the clinical application of caloric testing, they allow exploration of the relationship between VOR and vertigo perception. The slow-phase velocity (SPV) of the induced nystagmus and the subjective vertigo were thought to be tightly linked, but some studies have shown that age is associated with diminished vertigo in the presence of normal VOR [3] and using a binary scoring system this dissociation of SPV and absence of subjective caloric response was reported as strongly associated with imbalance in the elderly [4]. A higher incidence of motion sickness in migraineurs is well recognized [5, 6] and there have been reports of increased caloric nausea and vomiting in vestibular migraine (VM) patients [7]. There is also evidence of heightened vestibular motion sensitivity in patients with mal de debarquement syndrome (MdDS) [8]. Putative benign central vestibular disorders MdDS and PPPD, which are characterized by motion intolerance, have been thought to share some attributes of VM [9, 10].

### Aim

We setout to examine the relationship between caloric-evoked nystagmus SPV and subjective vertigo. Our hypothesis was that both would be increased in the putative central vestibular disorders of VM, MdDS, and PPPD, compared with peripheral labyrinthine disorders such as Meniere’s disease (MD), benign paroxysmal positioning vertigo (BPPV), other unilateral vestibular diseases (UVD) and non-vestibular dizziness (NVD). These NVD patients were referred to the clinic due to their dizzy or imbalance symptoms but their final diagnosis was not a vestibular disorder, (e.g., orthostatic hypotension, vasovagal syncope, and dizziness secondary to extrapyramidal conditions). We then investigated whether the caloric test subjective responses can assist in separation of the two commonest causes of recurrent spontaneous vertigo, VM, and MD.

## Methods

We included all patients who attended a neuro-otology clinic between January 2017 and December 2020, in whom interictal vestibular function testing had been undertaken prior to a history, examination and diagnosis by an expert neuro-otologist (SRDW), using Barany Society Criteria. Exclusions in the study were patients who could not provide consent and those patients who had testing without a clinical consultation. A single experienced operator (IH) performed all testing. Anxiety was not systematically assessed in this cohort of patients.

### The caloric test

Our caloric test irrigations were performed using a water stimulator, HORTMANN Aquamatic II* from GN Otometrics, Australia. A bithermal stimulation was delivered by irrigation with water warm 44 °C and cool 30 °C. Testing was performed with the patient lying down and their head elevated to 30°. Stimulus order and duration (25–40 s) varied in accord with usual clinical practice. If strong subjective responses were reported by the patient and the testing was poorly tolerated, the stimulus time was reduced and some patients received only single temperature irrigation. There are reports suggesting that monothermal caloric stimulation is adequate examination for many patients [11–13]. Following each ear irrigation, the mean SPV was measured using monocular infrared video-oculography camera and software ‘Visual Eyes’ from Micromedical, Chatham, Illinois, USA.

Caloric testing uses a non-physiologic stimulus which induces endolymphatic flow, as a temperature gradient from one side of the lateral semicircular canal to the other produces an endolymph response which is most likely due to changes in its specific gravity [14]. This leads to bending of the cupula and the embedded hair cells within the canal. The lateral semicircular canals are those most stimulated with the standard configuration of the caloric test [14]. The caloric stimuli are not calibrated [15] and, depending on the size and shape of the individual external ear canal, it is likely the stimulus strength can vary from person to person. However, for each patient, equal temperature and equal duration of stimulation is administered to each ear. If both lateral semicircular canals are normal, then the magnitude of their objective slow-phase velocity (SPV) responses are expected to be approximately the same.

The test result is obtained by comparing the objective SPV from each side using Jongkees formula [16] and this creates a single value, a percentage loss of unilateral function in the lateral semicircular canal. Our laboratory uses a value of > 25% canal paresis (CP) to determine unilateral abnormality on the caloric test. Where only binaural monothermal stimulation was possible, a simpler formula was used and the same abnormal value of CP > 25% was applied (Right SPV − Left SPV/Total × 100).

### Subjective responses

We developed a simple scoring process for perceived self-motion, the subjective vertigo score (SVS) and subjective nausea score (SNS). Following the first irrigation and without suggestion, the patient was asked to rate their induced ‘dizzy’ sensation, and this was recorded on a 0–4 scale (0 = no sensation, 1 = non-specific dizziness with no rotation, 2 = mild rotation, 3 = moderate rotation, 4 = severe rotation—often equated to their worst ever episode). They also rate their subjective nausea (0 = no nausea, 1 = mild, 2 = moderate, 3 = severe). The patient could compare these induced sensations to their ‘usual’ symptoms. This process was repeated following each subsequent irrigation. The clinical nurse (IH) recorded all responses. Other specific clinical parameters recorded at testing time were any history of migraine or/and motion sickness. The patient talking about their subjective sensations has the added advantage of maintaining patient alertness during the recording phase of the test. (Our subjective recording form is available in supplement documents).

We analyzed the nystagmus SPV, SVS, and SNS recording of the first warm irrigation 44 °C for all patients in all diagnostic groups. Then, in the patients who were able to complete a binaural bithermal test, we analyzed the same three variables from one warm 44 °C and one cool 30 °C irrigation from the same ear, on each patient. The side used was randomly selected except for the MD group, and in unilateral vestibular disease (UVD) patients, we used responses from their unaffected ears. To help separate the VM patients from those suffering MD, we employed machine learning (ML) models aiming to build a novel instrument for possible clinical use.

### Statistical analyses

Our analyses initially included all tests and then only those with complete data. Statistical tests were performed on IBM SPSS Statistics for Windows, Version 27.0. Armonk, NY. ANOVA was used to compare the SPV, SVS, and SNS between and within the disease groups. We used Pearson correlation test to compare the SPV with SVS within each group.

## Results

We collected data from 1373 consecutive patients (858 female and 515 male), mean age 57 ± 17 years. The breakdown in terms of diagnoses is shown in Table 1. There were 29 patients with bilateral vestibular loss who we excluded from any further analyses in this study. We had a total of 287 incomplete tests, which is a monothermal test (*n* = 274) or only one irrigation tolerated (*n* = 13). That is 21% of the total patient group could not complete the caloric test due to severe nausea or distress from the vestibular sensation. Table 1 shows that these were distributed unevenly across the diagnoses. The highest numbers of incomplete tests were in patients with VM and other putative benign central disorders.

**Table 1:** Total cohort caloric test demographics, disease groups and mean values of one warm irrigation

Diagnosis	No. of patients *n*	Mean age years ± SD	Gender M/F	Incomplete tests, *n* %		Warm irrigation mean SPV (°/s ± SD)	Warm irrigation mean SVS ± SD
VM definite or probable	553	50 ± 17	163/390	189	34	32 ± 14	2.8 ± 1.1
MdDS	27	49 ± 15	7/20	10	37	33 ± 13.6	2.9 ± 1.1
PPPD	15	59 ± 15	3/12	9	60	34 ± 18.1	3.1 ± 1.4
MD definite or probable	216	61 ± 16	109/107	7	3.2	30 ± 15.4	1.3 ± 1.4
UVD	72	61 ± 17	36/36	1	1.4	32 ± 13.0	1.7 ± 1.3
BPPV	270	65 ± 13	98/172	46	18	28 ± 14.0	1.8 ± 1.3
NVD	191	62 ± 17	85/106	25	13	30 ± 14.7	1.7 ± 1.5
BVL	29	65 ± 17	16/13	0	0		
Total	1373	57 ± 18	515/858	287	21		

*VM* vestibular migraine, *MdDS* Mal de Debarquement, *PPPD* Persistent postural perceptual dizziness, *MD* Meniere’s disease, *UVD* unilateral vestibular disorder, *BPPV* benign paroxysmal positioning vertigo, *NVD* non-vestibular disease, *BVL* Bilateral vestibular loss, *M* male, *F* female

The first irrigation for our patients was 44 °C and Table 1 illustrates nystagmus SPV and SVS mean values of one ear response or the non-affected side for MD and UVD patients (*n* = 1344). Initial ANOVA of the SVS, SNS, and SPV between the diagnostic groups showed there was a significant difference between the groups for the SVS (*F* = 50.9, *p* < 0.001) and the SNS (*F* = 20.9, *p* < 0.001). Then, the post hoc Bonferroni test showed there was no difference for either SVS or SNS between the VM, MdDS, and PPPD groups (*p* = 1.0) but a significant difference (*p* < 0.001) for both SVS and SNS when VM is compared to MD, BPPV, UVD, and the NVD patients. The SPV analysis also showed a significant difference between the diagnoses (*F* = 2.9, *p* = 0.02) but post hoc analysis showed this SPV difference was only between the BPPV patients and VM patients (*p* = 0.052), as BPPV mean SPV was less than VM (Table 1). There was no significance difference of SPV between VM and the MD, UVD, MdDS, PPPD or NVD groups (*p* = 1.0 for each).

The number of patients who completed the bithermal test was 1057, mean age 58 years (range 12–92). All demographic details are shown in Table 2 along with the mean SVS and SNS for each disease group and the mean SPV of induced nystagmus from two irrigations (one 44° and one 30°, see “Methods”).

**Table 2:** Demographics and data of one 30 °C and one 44 °C irrigation, from the patients who completed the test

Diagnosis	Completed tests, *n*	Mean age ± SD years	Gender M/F	Mean SPV ± SD deg/s		Mean SVS ± SD		Mean nausea score ± SD		Migraine headache *n* (%)	Motion sickness *n* (%)
				30 °C	44 °C	30 °C	44 °C	30 °C	44 °C		
VM definite or probable	366	50 ± 18	113/253	24 ± 8.9	29 ± 12.8	2.2 ± 1.1	2.5 ± 1.1	0.6 ± 0.8	0.8 ± 0.9	317 (87)	200 (55)
MdDS	17	51 ± 15	5/12	27 ± 13.6	31 ± 9.5	2.3 ± 1.4	2.6 ± 1.2	0.6 ± 1.0	0.6 ± 0.7	8 (47)	5 (29)
PPPD^a^	6^a^	54 ± 14	1/5	27 ± 10.6	30 ± 15.2	1.2 ± 0.7	2.0 ± 0.6	0.2 ± 0.4	0.3 ± 0.5	0	1 (17)
MD definite or probable	209	61 ± 15	105/104	22 ± 10.0	30 ± 15.4	1.1 ± 1.2	1.5 ± 1.3	0.1 ± 0.4	0.2 ± 0.5	64 (31)	47 (23)
UVD	71	61 ± 17	36/35	22 ± 11.2	31 ± 13.7	1.0 ± 0.6	1.7 ± 1.3	0.2 ± 0.5	0.1 ± 0.4	19 (26)	12 (17)
BPPV	224	65 ± 13	89/135	20 ± 10.2	27 ± 14.1	1.2 ± 1.1	1.6 ± 1.2	0.2 ± 0.5	0.3 ± 0.6	82 (38)	57 (26)
NVD	164	63 ± 17	76/88	25 ± 12.8	28 ± 14.2	1.1 ± 1.2	1.5 ± 1.4	0.2 ± 0.4	0.2 ± 0.5	51 (31)	39 (24)
Total	1057	58 ± 17	425/632	23 ± 10.0	30 ± 14.9	1.5 ± 1.3	1.8 ± 1.4	0.2 ± 0.5	0.3 ± 0.7	541 (51)	361 (34)

^a^PPPD had only 6 patients who completed the test

Age was not significantly correlated with SPV but was significantly correlated with the SVS (*r* = − 0.47, *p* < 0.001) and SNS (*r* = − 0.18, *p* < 0.001). Age was, therefore, entered as a covariate in all the following analyses.

**Fig. 1:**
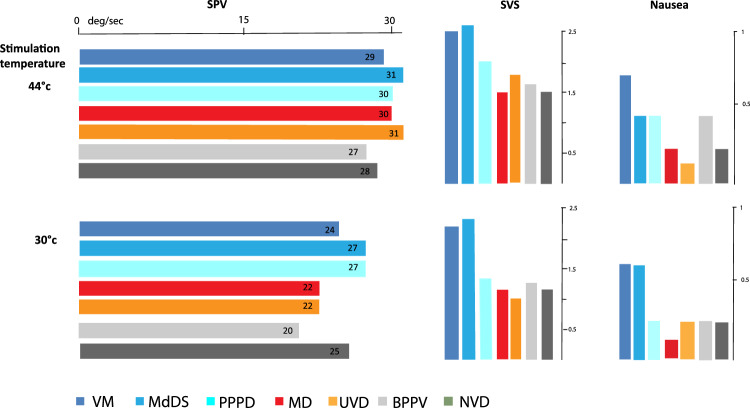
The mean SPV of caloric induced nystagmus for each diagnostic group is shown in horizontal bars. We have used one warm (44 deg) irrigation (*n* = 1344) and one cool (30 deg) irrigation (*n* = 1057), from the same ear of each patient. Included is the unaffected ear of the MD and UVD patients and random side selection for all others. The mean subjective scores (SVS) and nausea responses reported by the patients, to those irrigations, are shown by the vertical bars

The mean SPV was remarkably similar for all the diagnostic groups (Fig. 1). Although ANOVA showed a significant difference between the diagnoses (*F* = 2.7, *p* = 0.01) in the completed test group (*n* = 1057), post hoc analysis again showed that this was due to BPPV patients having lower SPV values than VM patients (*p* = 0.013). There were no other significant differences between the groups.

The significant difference across diagnostic groups in terms of SVS was (*F* = 65.4, *p* < 0.001). Post hoc tests showed that this difference was due to VM patients having greater scores than MD, BPPV, UVD, and NVD groups (*p* < 0.001 for all). MdDS was similar to VM; since PPPD had only six patients, we did not include this group in statistical analyses.

As shown in Table 2, SNS demonstrated similar trends to the SVS. There was a significant difference across groups (*F* = 12.5, *p* < 0.001). Again, VM patients scored significantly higher than patients with MD, BPPV, UVD, and NVD (*p* < 0.002 for all). There were no significant differences between MdDS (*n* = 17) and VM.

There were significant differences in the prevalence of motion sickness between diagnostic groups (Table 2, *F* = 19.7, *p* < 0.001); post hoc tests showed that this difference was due to VM patients having higher numbers than all the other groups (*p* < 0.001 for all).

### Patients with or without migraine headache

We separated patients within each diagnostic group, except VM, according to whether they had a history of migraine headache or not (Table 2). We first tested the VM group SVS against the migraineurs from all other diagnostic groups and found a significant difference (*F* = 19.3, *p* < 0.001) and post hoc tests showed that VM SVS was significantly different to the migraine patients in the other diagnostic groups (*p* < 0.001), except MdDS (*n* = 7). The VM patients scored a higher perception of test-induced vertigo compared to all other migraine patients. Nausea scores also showed a significant difference between VM and other reported migraineurs (*F* = 3.1, *p* = 0.008) but this difference was only with the BPPV and NVD groups (*p* = 0.5 for both).

We then compared patients across all diagnostic groups, to test whether patients with migraine headache were more sensitive to caloric stimulation (VM patients excluded). We focused on the main effect of migraine and the interaction between migraine and diagnosis. For SPV, there was no significant main effect of migraine and no interaction between migraine and diagnosis. In contrast, for SVS, there was a significant effect of migraine (*F* = 5.6, *p* = 0.02), whereby patients with history of migraine headache had higher SVS scores (3.5 ± 2.23) than those without (2.1 ± 2.05). There was no significant interaction between diagnosis and migraine. However, separate t-tests performed on the SVS between the migraine and no migraine patients, within each disease group, were significant, for MD (*t* = 3.1, *p* = 0.003), BPPV (*t* = 2.0, *p* = 0.05) and UVD (*t* = 3.3, *p* = 0.002) but not significant for NVD or the small MdDS group.

For nausea, there was a significant main effect of migraine (*F* = 9.4, *p* = 0.002) and a significant interaction between diagnosis and migraine (*F* = 2.4, *p* = 0.05). Post hoc *t* tests showed that patients with a history of migraine had higher SNS than those without migraine in the MD group (*t* = 3.8, *p* < 0.001) and UVD group (*t* = 2.9, *p* < 0.001) but not the other diagnostic groups.

Comparing our VM patient group against all other diagnosis-with-migraine patients, we discovered a significant difference between them, for SVS (*F* = 18.2, *p* < 0.001) and SNS (*F* = 3.1, *p* = 0.008), as the VM patients scored higher on both.

### Separation of VM and MD

**Fig. 2:**
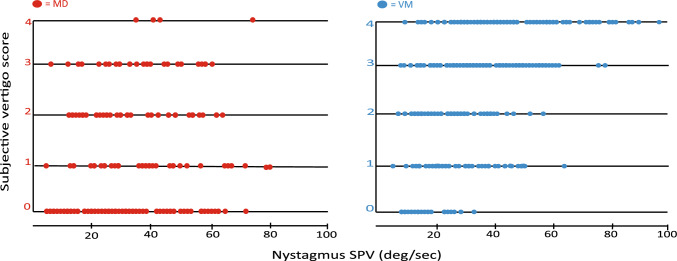
Two scatter plots showing the caloric test nystagmus SPV (deg/s) responses and the SVS response from a 44 °C irrigation for each patient, in the MD group (unaffected side) and the VM group. The spread of the nystagmus SPV is not dissimilar but the VM patients reported more severe vertigo showing higher SVS compared to the MD patients

The scatter plots in Fig. 2 show the SPV and SVS from one warm irrigation, for all patients with MD (unaffected side) and VM in the study, and it clearly contrasts the heightened subjective responses in the VM patients compared to the MD patients, across the range of SPV values. It also shows there was a large number of patients reporting no subjective responses during testing, and the majority being MD patients unaffected side (*n* = 47), compared to the VM group (*n* = 16). The mean age of the MD patients was slightly higher than the VM patients, 61 ± 18 years versus 50 ± 15 years and not expectedly, 76% of our MD patients were > 50 years old (*n* = 164) compared to 50% of the VM patient group (*n* = 277). Correlations between the nystagmus SPV and the SVS was stronger in the MD group than in the VM patients (*r* = 0.46 vs *r* = 0.32) with significance of *p* < 0.001 in both groups.

**Fig. 3:**
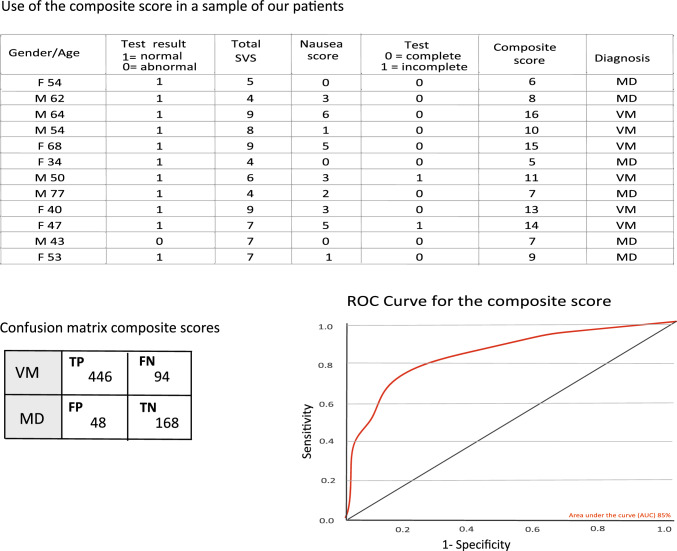
Example of using the composite score value in a selection of our patients. The ROC curve and the confusion matrix were created from the composite scores of all MD and the VM patients in the study. The confusion matrix values; *TP *true positive, *FN *false negative, *FP *false positive, *TN *true negative

We computed a composite score representing the sum of subjective scores collected during caloric testing (total SVS, 0–16) and total SNS, 0–12), binary values of test completion (1 = incomplete, 0 = complete) and the objective caloric test result (normal = 1, abnormal = 0) for each patient. The maximum achievable composite score was 30 but due to severe nausea, and this maximum was never reached. The average composite score for VM was 12 ± 4.3 and for MD 6 ± 4.5. Creating a ROC curve, and using a cut off composite score of 10, we sought to separate VM from MD with a sensitivity of 83% and a specificity of 71% (Fig. 3). A sample of our study patients in Fig. 3 shows how this might work.

### Machine learning modeling

We considered the possibility that ML models may surpass our composite metric in separating VM and MD, since the composite metric assigned equal importance to all collected data items.

### Methods used in ML

**Fig. 4:**
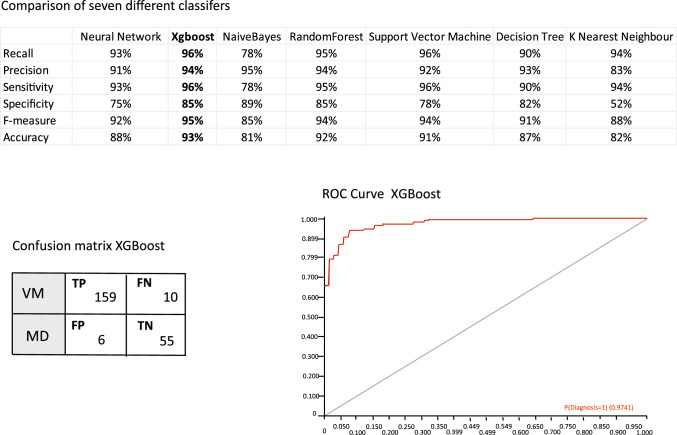
Comparative study of different machine learning classifiers with the metrics of accuracy, precision, sensitivity, *F*1-score, Specificity, and Recall. The ROC curve and confusion matrix from the best performing model, XGBoost (the ML confusion matrix shows 30% of the study numbers)

Patients with clinically probable or definite MD (*n* = 216) and patients with clinically probable or definite VM (*n* = 550) were selected from our cohort. Twenty “features” or variables including the induced nystagmus SVP (4 variables), % canal paresis, duration of irrigation, normal/abnormal test outcome, test completion status, SVS (4 variables) and SNS (4 variables), gender, age, history of migraine headache and history of motion sickness were used. We conducted a comparative analysis of seven machine learning (ML) techniques: Neural Network, XGBoost, Naive Bayes, Random Forest, Support Vector Machine (SVM), Decision Tree, and K Nearest Neighbors (KNN). We evaluated the performance of these techniques using various metrics, including Precision, Sensitivity, Specificity, F-score, and Accuracy. The dataset was split into a training set comprising 80% of the data and a testing set comprising the remaining 20%. The results obtained for each metric are shown in Fig. 4.

### Results of ML

Among the ML techniques, XGBoost consistently achieved high scores across multiple evaluation metrics. It demonstrated the highest Precision, Sensitivity, F-score, and Accuracy, indicating its effectiveness in correctly classifying instances of VM and MD (93% accuracy, 96% sensitivity and 85% specificity). XGBoost’s superior performance can be attributed to its ability to minimize false positives and false negatives while maximizing true positive and true negative predictions.

Naive Bayes also performed well in terms of Precision and Specificity, indicating its competence in correctly identifying positive and negative instances. However, it exhibited relatively lower scores in terms of Sensitivity and F-score, suggesting a higher likelihood of false negatives. On the other hand, KNN showed lower scores across all metrics, implying its limited effectiveness in accurately classifying VM and MD instances. The low Specificity score for KNN indicates a higher rate of false positives, which could lead to misdiagnosis.

It is important to consider the trade-off between different evaluation metrics based on the specific requirements of the application. For example, if minimizing false negatives is crucial, then models with high Sensitivity, such as XGBoost, would be preferred. Similarly, if avoiding false positives is a priority, models with high Precision and Specificity, such as XGBoost and Naive Bayes, could be more suitable.

## Discussion

We examined subjective and objective responses to caloric stimulation in many patients, across common vestibular disorders. Our results show that patients with VM and other putative central disorders experience greater sensations of vertigo and nausea with the caloric stimulation when compared with patients diagnosed with peripheral vestibular disorders and non-vestibular dizziness; in contrast, nystagmus slow-phase velocity (unaffected ear) was not significantly different between the patient groups.

### Previous work on subjective caloric responses

The value of collecting subjective patient responses during caloric testing has been reported in a few previous studies. Gibson et al., hypothesized that noting the subject’s sensations during bithermal caloric testing could possibly assist clinicians with separation of cerebellar disorders, peripheral vestibular disorders and anxiety [17]. In a retrospective study, Vitkovic et al., found the incompletion of the test due to patients reporting severe nausea was greater in the VM patients compared to migraine and nonmigraine-related dizziness patients [7]. Eliciting a nausea response with caloric testing was found to be more common in migraine patients and patients with motion sickness than patients without migraine [18], with suggestion that this is due to the association of motion sensitivity and migraine. Moran et al., asked study subjects (*n* = 63), to rank their perceptions of nausea and motion during caloric stimulation on a scale of 1–10. They reported the perception of nausea was a significantly distinguishing factor between VM patients and other vestibular disorders or migraineurs [19]. That study also found a weak to moderate significant relationship between the SPV and perception of motion. Unlike our findings, they report no significant difference of caloric perceived motion between the groups [19], but they do note that their stimulus (air) may not have been enough to elicit strong motion sensations.

The association of decreased vertigo and increased age in the presence of normal VOR has been reported by a few studies [3, 4] and similarly in our study, age was significantly correlated with the SVS and SNS and was entered as a covariate in our statistical analysis. Until now, a structured assessment of subjective caloric response in a very large cohort of patients during water caloric testing has not been undertaken.

In the adult population, vestibular migraine is the most common neurological cause of spontaneous vertigo [20, 21] and correct diagnosis is guided by diagnostic criteria such as those of Lempert et al., 2012 and more recently those of the Bárány Society and the International Headache Society [22]. The experience and clinical judgment of the physician remains critical as many other vestibular disorders have features that overlap with VM, with or without headache [23–25], and currently there is no pathognomonic test result to secure the VM diagnosis.

### Feasibility

We first used a numeric scale to express subjective vertigo and nausea, which required no additional time or resources and enabled the patient to compare their ‘usual dizzy’ symptoms to unilateral vestibular stimulation. When seeking to separate vestibular migraine from Meniere’s Disease, we found that the subjective scores complemented the objective SPV measures. We propose creating a tool using a composite score of all data collected at caloric testing time, which could help clinicians with separation of VM and MD. Utilizing ML models, we obtained a 96% sensitivity and 85% specificity. We hypothesize that even higher accuracies may be achieved by adding history and audiometry to the dataset used in this study.

### Possible mechanisms underlying caloric sensitivity

The pathophysiology of VM remains unclear, but has very reasonably been hypothesized to be similar to that of migraine with and without aura, which is currently considered a neurogenic disorder of sensitivity [26], with altered modulation of normal sensory stimuli with wide ramifications for central nervous system function [27–30]. Most likely, there is a genetic predisposition to migraine [31, 32]. Clinicians and researchers have long described symptoms of sensory sensitivity (e.g., photophobia and phonophobia), in migraine headache patients [5, 29, 33–35] and evidence suggests migraineurs show sensory dysmodulation with diminished sensory thresholds [36–39]. Abnormal visual processing and interictal habituation deficit have been reported on visual-evoked potential studies of migraine headache patients [40–44], also in VM patients [45]. Motion hypersensitivity and motion sickness is well reported in VM [46–51] and reduced vestibular perceptual thresholds in VM have been demonstrated with head movements [52] and using chair rotation [53]. Roll-tilt thresholds in VM patients compared to control group and migraine group is reduced [54, 55], suggesting changes in the central integration of canal and otolith signals and demonstrating a change in vestibular perception in VM that is unaccompanied by changes in vestibular-mediated eye movements [55]. Overall, our own research and that of previous investigators point to reduced vestibular perceptual threshold in migraine patients, including VM. Our findings also indicate that this perceptual hypersensitivity is greater in VM than in migraine headache without VM.

### Caloric stimulus intolerance

Our study demonstrated caloric stimulus intolerance in VM and other putative benign central disorders. Owing to this intolerance, only monothermal testing was possible for 34% of the VM group, 32% of the MdDS group, yet only 3.2% in the MD group. When we separated the non-VM diagnostic groups into patients with or without coexisting migraine headache, we found that within their diagnostic group, patients with migraine headache were more sensitive to caloric stimulation than those without migraine but patients with diagnoses of VM reported greater sensitivity to caloric stimulation than all other diagnosis group with coexisting migraine headaches.

Although our hypothesis was that both nystagmus SPV and subjective responses to caloric stimulation would be increased in the putative central vestibular disorders compared with peripheral labyrinthine disorders and NVD, we found that only the subjective responses demonstrated higher scores in VM. The seemingly dissociated relationship of ocular and perceptual responses to vestibular stimulation suggests that ascending vestibular signals are processed differently in VM and this might be a pathophysiological and clinical signature of this condition. Possibly, this provides evidence of abnormal sensory modulation which, as already noted, is believed to be a factor in migraine headache pathogenesis [26, 27]. It also shows that VOR and perception can be dissociated, i.e., pathways between vestibular afferents and VOR or cortical perception can be dissociated [56].

The primary outcome measure of the caloric test is a canal paresis which is based on the SPV response from one side compared to the other, after equal thermal stimulation. The normal vestibular peripheral system will respond with symmetrically SPV or within a range of normal < 25% difference in our clinic. Therefore, the presence of abnormality on caloric tests is expected to be more common in the peripheral disorders such as MD patients [57, 58]. However, normal results do not assure exclusion of MD, with normal caloric test results reported in up to 35% of MD patients [59–61].

Conventional caloric test result focuses on the SPV of the VOR but as there is evidence that vestibular perception and the eye movements are generated by mechanisms and pathways that are in part separate [55, 57, 62], we recommend additional recording of the subjective response (SVS), during the caloric test to extract further useful clinical information. In this study, the SVS showed a statistically significant difference between VM and MD (*p* < 0.001). Our findings emphasize the ongoing and increasing relevance of Barany’s foundational clinical investigation, even in the era of MRI, vHIT and VEMP. It also provides insight into the pathophysiology of VM. Comparable results were found for MdDS suggesting that they share perceptual vestibular hypersensitivity.

### Limitations of this study

Data collection was undertaken by a single operator at a single site and would need to be replicated in more locations. Clinical assessment was undertaken after the caloric test had been performed and the clinician was unblinded, therefore, caloric results could have influenced diagnosis. Our study had no systematic measurement of the anxiety that can be associated with caloric induced symptoms or of background anxiety that might have contributed to perceptual hypersensitivity. Prospective studies with measurement of anxiety and clinical assessment blinded to caloric results would help validate our findings.

## Conclusion

Assessment of caloric vertigo by verbal rating can be effortlessly incorporated into routine clinical testing. Patients classified with a putative central disorder reported greater perceived vertigo than those with a peripheral disorder. This significant perceptual difference indicates that subjective rating of vertigo in this context is a meaningful measure. We propose that when seeking to differentiate VM from MD, combining objective and subjective measures could provide optimal separation.

### Supplementary Information

Below is the link to the electronic supplementary material.

The form used by the clinician to collect the patient subjective responses during the caloric testing (DOCX 51 KB)

## Data Availability

Anonymized data will be shared by request from any qualified investigator.
