# Supplementary material for: Subjective and objective responses to caloric stimulation help separate vestibular migraine from other vestibular disorders
Source: J Neurol. 2023 Oct 17;271(2):887–98. doi: 10.1007/s00415-023-12027-z (PMC10828018; doi:10.1007/s00415-023-12027-z)
Supplement: Supplementary file 1 — The form used by the clinician to collect the patient subjective responses during the caloric testing (DOCX 51 KB) [file 415_2023_12027_MOESM1_ESM.docx]

**AGE: M/F**

**VERTIGO 30℃ (L) 30℃(R) 44℃ (L) 44℃(R)**

1. **No dizziness at all**
2. **Non-specific, not spinning**
3. **Mild Spinning**
4. **Moderate Spinning**
5. **Severe, worst ever**

**NAUSEA**

1. **Nil**

1. **Mild Nausea**
2. **Moderate Nausea**
3. **Severe Nausea**

**Migraine Hx: Motion sickness:**

**Comment:**

**Duration:**
